# Markers and risk factors for chronic kidney disease in sub-Saharan Africans: baseline levels and 12-month trajectories in newly referred patients in Cameroon

**DOI:** 10.1186/s12882-020-01760-6

**Published:** 2020-03-18

**Authors:** Halle Marie Patrice, Kengne Andre Pascal, Kaze Folefack François, Djantio Hilaire, Doualla Marie Solange, Ashuntantang Enow Gloria, Choukem Siméon Pierre

**Affiliations:** 1 Department of internal medicine Douala General Hospital Cameroon Faculty of medicine and pharmaceutical science, University of Douala, Douala, Cameroon; 2 Non-Communicable Diseases Research Unit, South African Medical Research Council, Cape Town, South Africa; 3 Faculty of medicine and biomedical sciences, University of Yaoundé I, Yaounde, Cameroon; 4 Higher Institute of Health Sciences, Université des Montagnes, Bangangte, Cameroon; 5 Department of internal medicine Douala General Hospital Cameroon, Faculty of medicine and pharmaceutical science, University of Douala, Douala, Cameroon; 6 Department of internal medicine Yaounde general hospital Cameroon, Faculty of medicine and biomedical sciences, University of Yaoundé I, Yaounde, Cameroon; 7 Department of Internal Medicine Douala General Hospital Cameroon, Faculty of Medicine and Pharmaceutical Sciences, University of Dschang, Dschang, Cameroon

**Keywords:** Risk factor, Chronic kidney disease, Trajectory, Cameroon

## Abstract

**Background:**

Little is known about the changes in disease makers and risk factors in patients with chronic kidney disease (CKD) under nephrological care in Africa. This study aimed to evaluate the baseline level of markers of CKD and their 12-month time-trend in newly referred patients in a tertiary hospital in Cameroon.

**Methods:**

This was a retrospective cohort study including 420 patients referred for CKD between 2006 and 2012 to the nephrology unit of the Douala General Hospital in the littoral region of Cameroon. Their disease and risk profile was assessed at baseline and every 3 months for 1 year. Estimated glomerular filtration rate (eGFR) was based on MDRD and Schwartz equations. CKD was diagnosed in the presence of eGFR< 60 ml/min/1.73 m^2^ and/or proteinuria> 1+ and/or abnormal renal ultrasound persisting for ≥3 months. Data analysis used mixed linear regressions.

**Results:**

Of the 420 patients included, 66.9% were men and mean age was 53.8 (15.1) years. At referral, 37.5% of the participants were at CKD Stage 3, 30.8% at stage 4 and 26.8% at stage 5. There was 168 (40%) diabetic and 319 (75.9%) hypertensive patients. After some improvement during the first 3 months, eGFR steadily decreased during the first year of follow-up, and this pattern was robust to adjustment for many confounders. Systolic and diastolic blood pressure levels significantly fluctuated during the first twelve months of follow-up. Changes in the levels of other risk factors and markers of disease severity over time were either borderline or non-significant.

**Conclusion:**

Patients with CKD in African settings are referred to the nephrologist at advanced stages. This likely translates into a less beneficial effects of specialised care on the course of the disease.

## Background

Chronic kidney disease (CKD) is a public health problem worldwide, with growing prevalence estimated at 11 to 13% in the adult population [1]. It has sustainably been a major contributor to the global burden of disease in the last two decades [2]. Main aetiologies of CKD worldwide are diabetes mellitus, hypertension and chronic glomerulonephritis [3]. CKD is characterized by 5 stages of irreversible impaired renal function, with progressive decline towards end stage kidney disease (ESKD) requiring renal replacement therapy (RRT). The rate of progression depends on comorbidities and risk factors. Effective strategies can slow the progression of CKD and may help reducing the risk of cardiovascular disease (CVD) and death [4, 5].

CKD disproportionately affects African descendants [6–8]. This is due to increased prevalence among Africans, of known risk factors for CKD such as diabetes, hypertension, genetic polymorphisms such as Apolipoprotein L1, and sickle cell trait [9–12]. CKD progresses more rapidly in people of African ethnicity [13–16]. In sub-Saharan Africa (SSA), CKD affects 12–23% adults [17–19], and mostly in their young and productive age [12, 20–23]. Despite the benefit of early referral on CKD progression, the rate of late referral of patients to the nephrologist is extremely high in SSA [24, 25], where access to RRT is limited [26]. Patients with CKD therefore face the problems of high out-of-pocket payment and poor outcome on RRT [27–29].

Few studies have reported the baseline profile of patients with CKD at referral and in SSA [23, 24, 28, 29]. Little is known on the evolution of their kidney function, related risk factors and markers of CKD progression under nephrological care. The objective of this study was to evaluate the baseline level of markers of CKD and their 12-month time-trend in newly referred patients at a tertiary hospital in Cameroon.

## Methods

### Study setting

This retrospective cohort study was based on registry and files of the out-patient section of the nephrology unit of the Douala General Hospital (DGH) in Cameroon. DGH is a 320-bedded public institution, serving as referral hospital for kidney disease for the Littoral region of the country and beyond. It has the largest haemodialysis unit of the country, and provides ongoing RRT to about 230 patients. The medical staff of the unit comprises two nephrologists, one general practitioner and twelve nurses. Patients with CKD referred to the unit are assigned a unique identifier and attached to one of the nephrologists, and then followed-up at intervals that are determined by the stage of the renal disease. At the first consultation in the unit, each patient has clinical assessment and laboratory tests done. The diagnosis of kidney disease was based on estimated glomerular filtration rate less than 60 ml/min and /or proteinuria. The aetiology of CKD was mostly based on clinical arguments. Patients are generally referred at the advanced stage of CKD when shrunken kidneys preclude any reliable histological diagnosis. Among those eventually eligible for such diagnosis, renal biopsy is seldom done in the unit. Ethical approval was obtained from the ethical committee board of the Douala University and administrative authorization from the DGH.

### Study participants

In the present study, we included all patients referred for CKD between January 2006 and December 2012. We did not include patients on renal replacement therapy in this study. Socio-demographic characteristics such as age and sex, and relevant clinical data including existing hypertension, diabetes mellitus, HIV, gout, and medication at referral were recorded. Blood pressure, aetiology of CKD, biological parameters including serum urea and creatinine level, glycaemia, uric acid, lipid profile, serum albumin and haemoglobin level were noted for the baseline level and every 3 months during the first 12 months of follow-up.

#### Definitions

The abbreviated version of the Modification of Diet in Renal Disease (MDRD) and Schwartz equations were used for estimated glomerular filtration rate (eGFR) in patients aged ≥18 Years and < 18 years respectively [30, 31]. CKD was defined by eGFR< 60 ml/min/1.73 m^2^ and/or proteinuria> 1+ and/or abnormal renal ultrasound (small shrunken, polycystic or asymmetric kidney), persisting for ≥3 months. Patients were classified following the Kidney Disease Improving Global Outcome (KDIGO) staging of CKD [32]. CKD stage 1: eGFR≧90 mL/min/1.73m^2^ with proteinuria or abnormal kidney, stage 2: 60≦eGFR< 90 mL/min/1.73m^2^ with proteinuria and /or abnormal kidney, stage 3: 30≦eGFR< 60 mL/min/1.73m^2^, stage 4: 15≦eGFR< 30 mL/min/1.73 m2, stage 5: eGFR< 15 mL/min/1.73m^2^. Diabetes was defined by a fasting serum glucose ≧126 mg/dL, or random glucose ≧200 mg/dL, HbA1c≧6.5%, or the use of hypoglycaemic agents. Hypertension at referral was defined as systolic blood pressure (SBP) ≥ 140 mmHg, diastolic blood pressure (DBP) ≥ 90 mmHg, or use of anti-hypertensive agents.

### Statistical analysis

Data analysis used SAS STAT v 9.1 for Windows® (SAS Institute Inc., Cary, NC, USA). We have reported baseline characteristics as count and percentages, and mean and standard deviation, and compared them across major subgroups via chi square tests and equivalents for qualitative variables, and Student’s t-test for continuous variables. Mixed linear regression models were used to examine changes in kidney function, determinants and indicators of disease complications (severity) during the first 12 months of follow-up while adjusting for baseline and changing levels of potential confounders during follow-up. Heterogeneity in the trajectories of key outcomes across major subgroups was investigated through interaction tests. Mixed linear models are suitable for handling longitudinal data with repeated measurements on continuous outcomes, particularly when there are missing data, which is rather a common situation in observational studies like this one. A *p*-value < 0.05 was used to indicate statistically significant results**.**

## Results

### Baseline characteristics of participants overall and by sex

A total of 420 patients were included; of whom 66.9% were men. The mean age (standard deviation) was 53.8 (15.1) years, with no significant difference in age between men and women (*p* = 0.09). The mean eGFR at referral was 28.6 (17.0) ml/min/1.73m^2^ overall, 30.9 (17.8) in men and 24.0 (14.2) ml/min/1.73m^2^ in women (*p* < 0.0001). The staging of kidney function at referral was: Stage 1 in 0.7% of participants, Stage 2 in 4.2%, Stage 3 in 37.5%, Stage 4 in 30.8% and Stage 5 in 26.8%; with a borderline significant difference by gender (*p* = 0.04). There was 168 (40%) diabetic, 319 (75.9%) hypertensive and 20 (4.9%) HIV positive patients at referral. Average blood pressure (BP) and creatinine levels were high, mostly similarly in men and women (all *p* > 0.39), while haemoglobin level was low, and much so in women (*p* = 0.0006) and the distribution of other hematologic parameters, electrolytes, lipid profile showed no major gender differences. Men were more likely to be smokers (*p* = 0.025), alcohol drinker (*p* < 0.0001), physically active (*p* = 0.001), to have gout (*p* = 0.011) and less likely to be HIV positive (*p* = 0.009) compared with women. Treatments, including for co-morbidities did not differ between men and women (Table 1).

**Table 1:** Baseline characteristics overall and by major subgroups

Characteristics	Overall	Men	Women	p	HTN	No HTN	P	DM	No DM	p
N (%)	420 (100)	281 (66.9)	139 (33.1)		319(76.0)	101 (24.0)		168 (40.0)	252 (60.0)	
Gender, men (%)	281 (66.9)	281 (100)	0 (0)		221 (69.3)	60 (59.4)	0.070	118 (70.2)	163 (64.7)	0.246
Age, years (SD)	53.8 (15.1)	54.7 (14.6)	52.1 (15.8)	0.097	57.9 (11.2)	41.1 (18.2)	< 0.0001	60.7 (8.2)	49.3 (16.6)	< 0.0001
Weight, kg (SD)	76.5 (16.3)	78.4 (17.7)	72.7 (12.5)	0.003	79.6 (14.5)	68.3 (18.1)	< 0.0001	78.1 (15.2)	75.4 (17.0)	0.206
SBP, mmHg (SD)	159 (31)	160 (30)	157 (34)	0.392	164 (30)	140 (27)	< 0.0001	162 (29)	157 (32)	0.087
DBP, mmHg (SD)	93 (18)	93 (18)	92 (18)	0.668	94 (19)	87 (16)	0.0004	89 (16)	96 (19)	0.0002
Urea	0.88 (0.56)	0.84 (0.52)	0.96 (0.62)	0.064	0.90 (0.53)	0.79 (0.61)	0.084	0.92 (0.50)	0.85 (0.60)	0.192
Creatinin	36.9 (27.4)	36.7 (27.6)	37.3 (27.2)	0.831	39.2 (28.7)	29.5 (21.2)	0.0004	35.5 (23.8)	37.9 (29.6)	0.367
eGFR	28.6 (17.0)	30.9 (17.8)	24.0 (14.2)	< 0.0001	26.9 (15.1)	34.9 (21.4)	0.001	27.8 (13.9)	29.2 (18.9)	0.387
Sodium	138.7 (10.4)	138.6 (10.6)	139.1 (9.8)	0.691	139.1 (11.4)	137.6 (5.7)	0.136	137.9 (14.8)	139.3 (5.8)	0.291
Potassium	4.5 (0.9)	4.5 (0.9)	4.6 (0.9)	0.355	4.6 (0.9)	4.5 (0.8)	0.586	4.7 (0.8)	4.4 (0.9)	0.002
Chrorine	103.8 (12.0)	103.8 (11.5)	103.8 (13.2)	0.970	104.2 (10.2)	102.5 (16.6)	0.426	102.8 (12.7)	104.4 (11.6)	0.250
Haemoglobin	10.7 (2.6)	11.0 (2.6)	10.0 (2.4)	0.0006	10.9 (2.5)	10.0 (2.7)	0.006	10.5 (2.1)	10.8 (2.8)	0.239
VGM	82.3 (11.9)	83.7 (7.5)	79.5 (17.3)	0.058	83.0 (10.2)	80.4 (14.5)	0.223	83.5 (9.9)	81.5 (13.0)	0.215
TCMH	28.3 (7.1)	27.9 (3.0)	29.0 (11.6)	0.461	27.8 (2.7)	29.5 (12.5)	0.311	27.7 (2.4)	28.6 (8.7)	0.317
Calcemia	88.5 (10.2)	88.6 (10.1)	88.3 (10.5)	0.865	88.6 (10.6)	87.9 (8.4)	0.553	89.0 (9.3)	88.1 (10.9)	0.443
Phosphate	46.8 (21.7)	46.7 (23.8)	47.0 (16.6)	0.913	47.8 (23.0)	42.1 (13.9)	0.043	45.0 (17.0)	48.1 (24.6)	0.291
Uric acid	86.2 (25.8)	86.5 (25.5)	85.2 (26.7)	0.700	87.3 (26.3)	80.2 (22.1)	0.097	82.5 (23.2)	89.2 (27.5)	0.028
Albumin	33.3 (13.7)	33.1 (14.5)	33.6 (12.6)	0.854	36.9 (14.5)	26.7 (9.2)	< 0.0001	34.9 (15.2)	32.4 (12.9)	0.345
Total cholesterol	2.3 (1.1)	2.2 (1.2)	2.4 (1.0)	0.264	2.1 (0.8)	2.8 (1.7)	0.002	2.1 (0.9)	2.3 (1.3)	0.322
HDL cholesterol	0.54 (0.37)	0.53 (0.36)	0.56 (0.40)	0.642	0.54 (0.39)	0.53 (0.27)	0.767	0.52 (0.32)	0.55 (0.41)	0.116
LDL cholesterol	1.4 (0.7)	1.4 (0.7)	1.4 (0.6)	0.653	1.4 (0.6)	1.5 (1.1)	0.653	1.1 (0.6)	1.4 (0.7)	0.556
Triglycerides	1.3 (0.9)	1.1 (0.7)	1.6 (1.1)	0.002	1.2 (0.7)	1.6 (1.4)	0.052	1.2 (0.7)	1.3 (1.0)	0.307
Diabetes, n (%)	168 (40.0)	118 (42.0)	50 (36.0)	0.236	147 (46.1)	21 (20.8)	< 0.0001	168 (100)	0 (0)	< 0.0001
Hypertension, n (%)	319 (75.9)	221 (78.6)	98 (70.5)	0.066	319 (100)	0 (0)	< 0.0001	147 (87.5)	172 (68.2)	< 0.0001
Smoking, n (%)	24/395 (6.1)	21/262 (8.0)	3/133 (2.3)	0.025	19/297 (6.4)	5/98 (5.1)	0.809	8/155 (5.2)	16/240 (6.7)	0.668
Alcohol, n (%)	129/388 (33.2)	110/258 (42.6)	19/130 (14.6)	< 0.0001	106/293 (36.2)	23/95 (24.2)	0.033	44/152 (28.9)	85/236 (36.0)	0.153
Sedentarity	163/358 (45.5)	95/240 (39.6)	68/118 (57.6)	0.001	134/269 (49.8)	29/89 (32.6)	0.005	69/140 (49.3)	94/218 (43.1)	0.277
HIV infection, n (%)	20/411 (4.9)	7/274 (2.5)	13/137 (9.5)	0.009	4/311 (1.3)	16/100 (16.0)	< 0.0001	3/165 (1.8)	17/246 (6.9)	0.043
Gout, n (%)	28/411 (6.8)	25/275 (9.1)	3/136 (2.2)	0.011	27/313 (8.6)	1/98 (1.0)	0.005	10/166 (6.0)	18/245 (7.3)	0.692
Stage CKD (*n* = 403)				0.040			0.002			0.013
1	3 (0.7)	3 (1.1)	0 (0)		1 (0.3)	2 (2.3)		0 (0)	3 (1.3)	
2	17 (4.2)	13 (4.8)	4 (3.0)		7 (2.2)	10 (11.4)		2 (1.2)	15 (6.4)	
3	151 (37.5)	111 (41.1)	40 (30.1)		116 (36.8)	35 (39.8)		70 (41.9)	81 (34.3)	
4	124 (30.8)	80 (29.6)	44 (33.1)		104 (33.0)	20 (22.7)		54 (32.3)	70 (29.7)	
5	108 (26.8)	63 (23.3)	45 (33.8)		87 (27.6)	21 (23.9)		41 (24.5)	67 (28.4)	
**Treatments**										
ACE inhibitors	314 (74.8)	123 (75.8)	101 (72.7)	0.551	263 (82.4)	51 (50.5)	< 0.0001	137 (81.5)	177 (70.2)	0.011
Loop diuretics	130 (30.9)	86 (30.6)	44 (31.6)	0.823	86 (27.0)	44 (43.6)	0.002	56 (33.3)	74 (29.4)	0.391
Calcium channels blockers	198 (47.1)	125 (44.5)	73 (52.5)	0.146	185 (58.0)	13 (12.9)	< 0.0001	81 (48.2)	117 (46.4)	0.765
Thiazide diuretic	206 (49.0)	142 (50.5)	64 (46.0)	0.408	177 (55.5)	29 (28.7)	< 0.0001	91 (54.2)	115 (45.6)	0.091
Antialdosterone	17 (4.0)	12 (4.3)	5 (3.6)	> 0.999	4 (1.2)	13 (12.9)	< 0.0001	4 (2.4)	13 (5.2)	0.208
ARB	17 (4.0)	10 (3.6)	7 (5.0)	0.445	14 (4.3)	3 (3.0)	0.773	11 (6.5)	6 (2.4)	0.043
Beta blockers	54 (12.9)	36 (12.8)	18 (12.9)	> 0.999	48 (15.0)	6 (5.9)	0.017	17 (10.1)	37 (14.7)	0.184
Central agent	20 (4.8)	14 (5.0)	6 (4.2)	> 0.999	20 (6.3)	0 (0)	0.006	9 (5.4)	11 (4.4)	0.647
Biguanide	6 (1.4)	4 (1.4)	2 (1.4)	> 0.999	6 (1.9)	0 (0)	0.343	6 (3.6)	0 (0)	0.004
Sulphonamides	57 (13.6)	41 (14.6)	16 (11.5)	0.450	50 (15.7)	7 (6.9)	0.029	57 (33.9)	0 (0)	< 0.0001
Glinides	1 (0.2)	1 (0.4)	0 (0)	> 0.999	1 (0.3)	0 (0)	> 0.999	1 (0.6)	0 (0)	0.400
Insulin	34 (8.1)	22 (7.8)	12 (8.6)	0.850	28 (8.8)	6 (5.9)	0.411	34 (20.2)	0 (0)	< 0.0001

*HTN* Hypertension, *DM* Diabetes mellitus, *SD* Standard deviation, *SBP* Systolic blood pressure, *DPB* Diastolic blood pressure, *eGFR* Estimated Glomerular filtration rate, *MCV* Mean corpuscular volume, *MCH* Mean corpuscular haemoglobin, *HDL* High density lipoprotein, *LDL* Low density lipoprotein, *HIV* Human immune-deficiency virus, *ACE* Angiotensin converting enzyme, *ARB* Angiotensinogen receptor blockers

### Baseline characteristics in other major subgroups of participants

Baseline differences were apparent between participants with diabetes and those without, with regard to age (*p* < 0.0001), diastolic blood pressure (*p* = 0.0002), potassium (*p* = 0.002), uric acid (*p* = 0.028), prevalent hypertension (*p* < 0.0001), stage of kidney function (*p* = 0.013), treatment with ACE inhibitors (*p* = 0.011) or ARA II (*p* = 0.043). Compared with participants without hypertension, those with hypertension were more likely to be older, to have higher weight, creatinine, haemoglobin, albumin and phosphate levels (all *p* < 0.043). They were also more likely to have diabetes (*p* < 0.0001), to be alcohol drinkers (*p* = 0.033), sedentary (*p* = 0.005), to comprise fewer people with HIV infection (*p* < 0.0001), to be referred with advanced stage CKD (*p* = 0.002).

### Trajectories of kidney function during follow-up

**Fig. 1:**
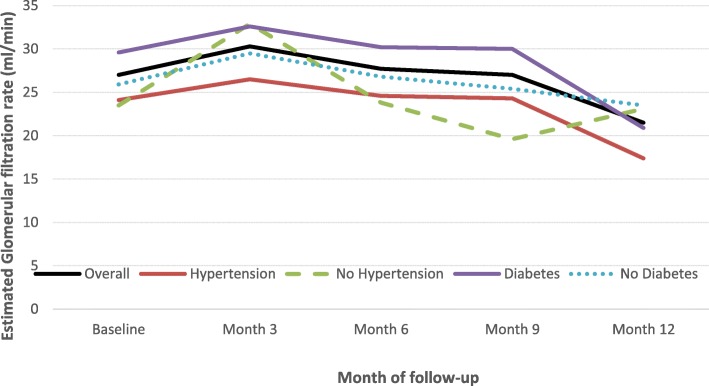
Trajectory of eGFR overall and by major subgroups. *P*-values for linear trends are 0.003 in the overall sample, 0.058 for participants with- and 0.013 for those without diabetes; 0.010 for participants with- and 0.030 for those without hypertension

After some improvement during the first 3 months of follow-up, eGFR steadily decreased during the first twelve months of follow-up, and this pattern was robust to adjustment for age, sex, status for hypertension, diabetes mellitus, smoking, alcohol consumption and HIV infection (*p* = 0.003); Table 2 and Fig. 1. In similar analyses stratified by baseline status for diabetes (Table 3) or hypertension (Table 4), the pattern was mostly similar with however a significant effect only in participants with diabetes (*p* = 0.013) but not in those without (*p* = 0.205). While there was no evidence of statistical interaction by diabetes status in the trajectories of eGFR (interaction *p* = 0.646), a borderline interaction was apparent in the effects by status for hypertension (*p* = 0.054), primarily driven by an improvement in the eGFR between 9 and 12 months of follow-up among participants without hypertension at baseline (Fig. 1). Serum urea level did not change significantly during follow-up overall and within major subgroups of participants.

**Table 2:** Trajectory of key variables in the overall sample

Variables	Baseline	Month 3	Month 6	Month 9	Month 12	*p*-value
Creatinine	41.7 (3.7)	41.5 (3.9)	47.7 (4.1)	52.1 (4.5)	59.1 (5.0)	0.0001
eGFR	27.0 (2.3)	30.3 (2.4)	27.7 (2.5)	27.0 (2.8)	21.5 (3.1)	0.003
Urea	1.00 (0.08)	0.97 (0.08)	1.04 (0.09)	1.01 (0.10)	1.21 (0.11)	0.178
SBP	154.4 (3.2)	142.1 (3.7)	147.3 (3.9)	145.5 (4.4)	156.0 (5.1)	< 0.0001
DBP	92.4 (2.0)	85.4 (2.3)	86.3 (2.4)	83.9 (2.7)	85.6 (3.1)	< 0.0001
Sodium	136.9 (1.2)	136.9 (1.5)	136.1 (1.7)	139.7 (2.0)	140.4 (2.4)	0.345
Potassium	4.59 (0.12)	4.55 (0.13)	4.43 (0.14)	4.67 (0.16)	4.67 (0.20)	0.382
Calcium	87.4 (1.5)	84.6 (2.2)	88.1 (1.9)	89.0 (2.6)	88.0 (2.8)	0.558
Haemoglobin	10.2 (0.6)	10.0 (0.7)	9.6 (0.7)	10.1 (0.8)	11.1 (0.9)	0.090
TCMH	30.2 (1.4)	30.4 (1.6)	32.2 (1.7)	31.4 (1.9)	32.0 (2.7)	0.535
VGM	82.1 (2.1)	78.8 (2.7)	85.9 (2.9)	84.9 (3.4)	91.0 (4.5)	0.051
Phosphate	45.4 (3.0)	40.7 (5.3)	41.9 (4.0)	45.9 (5.5)	40.4 (5.3)	0.624
Uric acid	78.3 (3.9)	67.6 (5.4)	70.1 (5.1)	68.3 (7.2)	65.4 (8.2)	0.034
Albumin	30.5 (2.4)	35.3 (3.0)	27.8 (3.3)	30.5 (4.1)	37.0 (7.3)	0.125
Total cholesterol	2.4 (0.2)	2.0 (0.2)	1.8 (0.3)	1.9 (0.3)	2.6 (0.4)	0.036
HDL cholesterol	0.51 (0.06)	0.36 (0.19)	0.30 (0.15)	0.44 (0.27)	0.53 (0.24)	0.685
Triglycerides	1.7 (0.2)	1.3 (0.4)	1.6 (0.3)	1.5 (0.4)	1.4 (0.4	0.648

Estimates are mean and standard error of the mean, and are adjusted for age, sex, diabetes, hypertension smoking alcohol consumption, HIV status *SBP* Systolic blood pressure, *DPB* Diastolic blood pressure, *eGFR* Estimated Glomerular filtration rate, *MCH* Mean corpuscular haemoglobin, *MCV* Mean corpuscular volume, *HDL* High density lipoprotein, *LDL* Low density lipoprotein

**Table 3:** Trajectory of key variables in participants with and without diabetes

Variables	Subgroup	Baseline	Month 3	Month 6	Month 9	Month 12	*p*-value	Interaction p
Creatinine	Diabetes	35.6 (8.3)	35.1 (8.4)	40.9 (8.7)	47.5 (9.2)	60.8 (9.7)	0.001	0.334
	No diabetes	42.8 (4.7)	43.1 (5.0)	49.7 (5.3)	52.8 (5.7)	53.5 (6.7)	0.058	
eGFR	Diabetes	29.6 (4.2)	32.6 (4.3)	30.2 (4.5)	30.0 (4.9)	20.9 (5.1)	0.013	0.646
	No diabetes	25.9 (2.8)	29.5 (3.1)	26.8 (3.2)	25.4 (3.6)	23.5 (4.2)	0.205	
Urea	Diabetes	1.13 (0.17)	1.07 (0.18)	1.09 (0.19)	1.11 (0.22)	1.34 (0.21)	0.531	0.862
	No diabetes	1.02 (0.10)	1.02 (0.10)	1.10 (0.11)	1.04 (0.12)	1.20 (0.15)	0.452	
SBP	Diabetes	153.1 (8.4)	145.6 (8.7)	150.8 (9.1)	162.6 (9.9)	149.4 (10.3)	0.030	0.006
	No diabetes	153.5 (3.6)	139.2 (8.3)	144.6 (4.6)	136.3 (5.0)	143.9 (6.4)	< 0.0001	
DBP	Diabetes	86.2 (8.4)	81.2 (4.6)	83.8 (4.9)	83.6 (5.5)	81.7 (5.6)	0.193	0.174
	No diabetes	96.9 (2.4)	88.9 (2.7)	88.9 (2.9)	85.2 (3.2)	88.7 (4.0)	< 0.0001	
Sodium	Diabetes	127.6 (3.9)	130.2 (4.1)	127.1 (4.4)	129.6 (5.1)	131.9 (5.2)	0.430	0.298
	No diabetes	138.8 (1.3)	136.4 (1.9)	137.7 (1.9)	141.6 (2.2)	141.7 (3.1)	0.256	
Potassium	Diabetes	4.8 (0.3)	4.8 (0.3)	4.6 (0.3)	4.9 (0.3)	5.0 (0.4)	0.542	0.935
	No diabetes	4.4 (0.1)	4.3 (0.2)	4.2 (0.2)	4.4 (0.2)	4.4 (0.3)	0.552	
Calcium	Diabetes	84.2 (4.0)	74.7 (4.8)	84.9 (4.7)	84.4 (5.7)	84.2 (5.4)	0.098	0.216
	No diabetes	88.9 (1.7)	89.1 (2.6)	88.9 (2.2)	91.9 (2.9)	90.4 (3.6)	0.848	
Haemoglobin	Diabetes	10.0 (0.7)	9.0 (0.9)	9.1 (1.1)	10.3 (1.5)	12 (1.2)	0.022	0.005
	No diabetes	11.2 (0.6)	10.7 (0.7)	10.3 (0.7)	10.0 (0.7)	9.8 (0.9)	0.079	
TCMH	Diabetes	27.2 (1.7)	27.4 (1.8)	28.5 (1.8)	28.2 (2.0)	28.7 (2.2)	0.582	0.981
	No diabetes	30.8 (1.8)	29.7 (2.5)	32.5 (2.6)	31.5 (3.1)	32.2 (5.9)	0.908	
VGM	Diabetes	84.2 (3.0)	85.3 (4.0)	93.1 (4.3)	90.3 (5.1)	91.8 (4.7)	0.069	0.325
	No diabetes	81.2 (2.6)	75.6 (3.5)	80.7 (3.9)	82.0 (4.4)	92.3 (8.3)	0.260	
Phosphate	Diabetes	47.3 (6.2)	52.1 (8.3)	43.2 (8.1)	50.7 (9.1)	48.3 (7.8)	0.707	0.586
	No diabetes	46.4 (4.4)	39.6 (7.2)	44.7 (5.7)	51.3 (7.5)	35.0 (9.0)	0.433	
Uric acid	Diabetes	73.3 (6.5)	66.1 (7.3)	69.1 (8.6)	72.3 (11.3)	78.0 (14.1)	0.431	0.407
	No diabetes	80.9 (4.5)	68.5 (7.2)	69.0 (6.2)	63.0 (8.9)	56.4 (9.8)	0.021	
Albumin	Diabetes	26.4 (2.9)	30.6 (4.5)	19.0 (6.9)	24.2 (13.3)	21.6 (13.3)	0.715	0.480
	No diabetes	33.0 (3.3)	38.1 (3.9)	31.2 (4.2)	33.7 (4.9)	48.9 (10.1)	0.150	
Total cholesterol	Diabetes	1.8 (0.3)	0.8 (0.4)	1.3 (0.4)	–	2.3 (0.4)	0.307	0.889
	No diabetes	2.6 (0.2)	2.3 (0.3)	1.9 (0.3)	2.1 (0.3)	2.6 (0.6)	0.182	
HDL cholesterol	Diabetes	0.34 (0.15)	0.15 (0.21)	0.21 (0.21)	–	0.28 (0.26)	0.626	0.852
	No diabetes	0.61 (0.08)	0.34 (0.46)	0.31 (0.22)	0.57 (0.33)	0.92 (0.48)	0.733	
Triglycerides	Diabetes	0.93 (0.21)	0.84 (0.30)	0.98 (0.32)	–	0.70 (0.40)	0.846	0.119
	No diabetes	2.01 (0.22)	−0.46 (0.48)	1.63 (0.50)	1.63 (0.41)	1.58 (0.57)	0.118	

Estimates are mean and standard error of the mean, and are adjusted for age, sex, diabetes, hypertension smoking alcohol consumption, HIV status *SBP* Systolic blood pressure, *DPB* Diastolic blood pressure, *eGFR* Estimated Glomerular filtration rate, *MCH* Mean corpuscular haemoglobin, *MCV* Mean corpuscular volume, *HDL* High density lipoprotein, *LDL* Low density lipoprotein

**Table 4:** Trajectory of key variables in participants with and without hypertension

Variables	Subgroup	Baseline	Month 3	Month 6	Month 9	Month 12	*p*-value	Interaction p
Creatinine	Hypertension	41.5 (6.9)	41.9 (7.1)	46.7 (7.2)	50.9 (7.4)	58.1 (7.8	0.0028	0.613
	No hypertension	44.7 (6.1)	41.7 (6.8)	55.0 (7.4)	59.7 (8.3)	64.3 (10.2)	0.042	
eGFR	Hypertension	24.1 (3.3)	26.5 (3.4)	24.6 (3.5)	24.3 (3.7)	17.4 (3.9)	0.010	0.054
	No hypertension	23.5 (3.6)	32.9 (4.3)	23.8 (5.0)	19.6 (6.0)	23.1 (6.9)	0.030	
Urea	Hypertension	1.01 (0.12)	1.06 (0.12)	1.04 (0.13)	1.00 (0.14)	1.22 (0.14)	0.253	0.273
	No hypertension	1.09 (0.13)	0.95 (0.14)	1.20 (0.16)	1.16 (0.24)	1.15 (0.24)	0.188	
SBP	Hypertension	163.9 (5.3)	151.0 (5.7)	156.1 (5.8)	159.8 (6.4)	153.5 (6.9)	0.0003	0.355
	No hypertension	138.9 (6.4)	130.9 (7.1)	135.0 (7.8)	125.1 (8.4)	138.1 (10.3)	0.099	
DBP	Hypertension	94.7 (3.2)	87.5 (3.5)	89.8 (3.6)	87.5 (3.9)	87.5 (4.1)	0.0002	0.713
	No hypertension	88.7 (3.6)	82.6 (4.0)	82.0 (4.6)	80.2 (4.8)	85.4 (5.9)	0.043	
Sodium	Hypertension	138.0 (2.4)	135.1 (2.7)	138.3 (2.9)	140.6 (3.2)	141.6 (3.7)	0.207	0.388
	No hypertension	138.4 (1.9)	139.8 (2.2)	135.3 (2.5)	141.5 (3.1)	141.9 (3.8)	0.221	
Potassium	Hypertension	4.54 (0.20)	4.54 (0.21)	4.31 (0.22)	4.53 (0.24)	4.59 (0.27)	0.241	0.011
	No hypertension	5.01 (0.24)	4.60 (0.30)	4.98 (0.33)	5.67(0.41)	5.08 (0.52)	0.154	
Calcium	Hypertension	88.7 (2.6)	83.2 (3.2)	89.8 (3.1)	89.4 (3.5)	90.0 (3.7)	0.171	0.402
	No hypertension	87.8 (2.5)	91.6 (3.6)	87.2 (3.4)	94.5 (5.3)	81.8 (6.1)	0.339	
Haemoglobin	Hypertension	10.8 (0.8)	10.2 (0.8)	9.9 (0.8)	9.7 (0.9)	10.5 (0.9)	0.100	0.058
	No hypertension	9.9 (0.8)	9.5 (1.0)	9.1 (2.1)	10.7 (2.5)	10.1 (1.9)	0.960	
TCMH	Hypertension	29.6 (1.6)	29.7 (1.9)	33.2 (1.9)	30.6 (2.3)	31.6 (3.0)	0.153	0.351
	No hypertension	28.8 (3.9)	29.1 (4.3)	23.4 (4.7)	26.3 (5.1)	28.4 (6.7)	0.424	
VGM	Hypertension	84.3 (3.3)	80.2 (3.8)	88.0 (4.0)	85.3 (4.7)	92.2 (6.1)	0.165	0.889
	No hypertension	77.8 (4.0)	75.2 (5.4)	81.6 (5.9)	83.2 (6.4)	92.2 (8.3)	0.425	
Phosphate	Hypertension	52.6 (5.0)	51.7 (6.5)	49.7 (5.8)	54.4 (6.9)	51.2 (7.0)	0.902	0.860
	No hypertension	44.3 (4.7)	37.4 (14.9)	40.5 (7.1)	67.6 (15.1)	34.7 (10.4)	0.516	
Uric acid	Hypertension	92.4 (6.2)	82.2 (7.3)	84.3 (7.1)	83.6 (8.7)	79.6 (9.4)	0.089	0.872
	No hypertension	72.5 (5.9)	59.0 (8.6)	71.9 (11.3)	65.2 (20.4)	–	0.347	
Albumin	Hypertension	33.8 (4.0)	30.6 (6.6)	30.9 (5.8)	28.2 (7.6)	27.1 (13.1)	0.825	0.246
	No hypertension	25.9 (2.8)	32.0 (3.4)	23.4 (4.3)	28.3 (5.1)	41.3 (9.9)	0.078	
Total cholesterol	Hypertension	2.41 (0.20)	2.00 (0.25)	1.87 (0.29)	1.96 (0.34)	2.66 (0.42)	0.114	0.916
	No hypertension	2.24 (0.33)	2.81 (0.78)	0.15 (1.19)	1.72 (0.96)	1.89 (0.86)	0.513	
HDL cholesterol	Hypertension	0.48 (0.12)	0.32 (0.17)	0.37 (0.17)	0.41 (0.33)	0.50 (0.22)	0.633	0.761
	No hypertension	0.57 (0.13)	–	-	0.00009 (0.35)	–	0.158	
Triglycerides	Hypertension	1.83 (0.13)	0.92 (0.34)	1.53 (0.31)	1.82 (0.39)	1.51 (0.34)	0.262	0.956
	No hypertension	1.32 (0.47)	–	–	1.83 (1.79)	–	Not computable	

Estimates are mean and standard error of the mean, and are adjusted for age, sex, diabetes, hypertension smoking alcohol consumption, HIV status *SBP* Systolic blood pressure, *DPB* Diastolic blood pressure, *eGFR* Estimated Glomerular filtration rate, *MCH* Mean corpuscular haemoglobin, *MCV* Mean corpuscular volume, *HDL* High density lipoprotein, *LDL* Low density lipoprotein, *NC* Not computable

### Trajectories of other markers and risk factors for CKD

Systolic and diastolic BP levels significantly fluctuated during the first twelve months of follow-up, with both ups and downs observed between consecutive visits (both *p* < 0.0001); (Table 2), with suggestions that these fluctuations occurred in a differential way for SBP between participants with diabetes and those without (interaction *p* = 0.006); (Table 3), but not for DBP (*p* = 0.174), nor by status for hypertension (both interaction *p* > 0.355); (Table 4). Haemoglobin levels decreased between baseline and 3-month visit, and steadily increased thereafter, although the overall effect was not significant (*p* = 0.09). This pattern was consistent by status hypertension, while differing trajectories were observed among participants with diabetes (significant increase over time, *p* = 0.022) and those without diabetes (borderline significant decreased over time, *p* = 0.079), with significant statistical interaction (interaction *p* = 0.005). Other haematological parameters, electrolytes and lipid profile did not change significantly during follow-up overall and within major subgroups of participants.((Tables 2, 3 and 4)

## Discussion

In this study, we have for the first time described the time-trend in the trajectory of kidney function, risk makers and health consequences in patients with CKD in a SSA setting. We found a deteriorating kidney function over time, which was robust to adjustment for potential confounders and broadly similar across levels of major risk factors such as diabetes mellitus and hypertension; although some late improvement was observed among non-hypertensive participants, resulting in borderline interaction by status for hypertension. We found fluctuating levels of blood pressure over time, which was significantly different by status for diabetes, but no for hypertension, and likely reflecting the difficulties to achieve and maintain adequate blood pressure control in patients with CKD. Changes in the levels of other risk factors and markers of disease severity over time were either borderline or non-significant.

In its natural history, CKD progresses silently to ESKD and studies have shown that trajectories of glomerular filtration rate (GFR) over time are heterogeneous [33–38]. The rate of decline in kidney function is related to the advancement of CKD stages, to risk factors for CKD progression, and treatments [15, 39–41]. In the present study, more than half of the population was at CKD Stage 4 (30.8%) and Stage 5 (26.8%) at referral, with men seemed to be referred to the service earlier than women. The main reason of this disparity is that women in this setting would mostly be in financial disadvantage for multiple reasons including non-employment, low income, dependence on the male partner. These are advanced stages of CKD with almost always inexorable progression to ESKD [42–44]. But this progression varies across populations and according to the presence of certain comorbidities [42, 43, 45, 46]. Morgan et al. reported a 1-year cumulative incidence of ESKD of 4.3% from CKD stage 4 and 49% from stage 5; and the level of proteinuria was the main predictor of the risk of progressing to ESKD with a median progression time of 9 months for participants with high proteinuria and 19 months for those with lower proteinuria [47]. A potential initial improvement of the kidney function in our sample, likely reflect the effects of treatments adjustment, intensification or initiation by nephrologists at the first visit. In general most patients with CKD in this setting would have been on non-optimal treatments prior to their referral to nephrologists.

Hypertension and diabetes are well-known risks factors for the development and progression of CKD [9, 40, 41, 48, 49]. Studies have reported that the rate of eGFR decline was significantly associated with mean blood pressure [50, 51]. Hemmelgarn et al. reported that the decline in eGFR after a 2-years follow-up, was highest among those with diabetes mellitus [52]. Trajectories of kidney function in our sample were mostly similar by diabetes and hypertension status. This likely reflected the challenges of controlling these major CKD risk factors, and not the lack of their effect on CKD progression. This is substantiated for instance by the similarity of the trajectories of systolic and diastolic blood pressure in participants with and without hypertension during follow-up. Hypertension is generally exacerbated in the context of CKD. Therefore, the parallel trajectories of BP levels in participants with and without hypertension in our sample, to some extent reflect the success of therapeutic measures to lower BP in our sub-sample with hypertension. At baseline, a very large proportion of our participants were on BP lowering medications, with ¾ on Renin angiotensin aldosterone system blockers (RAAS) witch beneficial effects are consistent in the literature [53, 54]. But in the absence of updated data on treatment during follow-up, it is not possible to determine if BP lowering treatments were appropriately intensified during follow-up in our sample.

We did not have data to assess the effects of glycemic control on the trajectory of kidney function in people with diabetes. However, the distribution of baseline glucose control treatment suggests that their intensity was likely not enough to achieve and maintain good glycemic control. Almost all participants with diabetes also had hypertension with differentially high uptake of reno protective drugs such as angiotensin converting enzyme (ACE) inhibitors and angiotensine receptor inhibitors among those with diabetes at baseline (in line with guidelines) [53, 55–57]. In general however, BP levels and SBP in particular are the stronger predictor of renal outcome in people with diabetes [57, 58]. Because of the late referral, biological perturbations related to CKD were already apparent in our sample at baseline including hematological abnormalities and low serum albumin. These abnormalities mostly persisted during follow-up although some late improvements were observed particularly for total hemoglobin levels in participants with diabetes, and serum albumin in the overall sample. In the absence of data on specific treatments targeting those attributes, we can speculate that the observed late improvement was likely due to the selection process. It is indeed an expectation that during follow-up participants with severe disease at baseline (advanced stage CKD) would fall out (through initiation of renal replacement therapy for instance), and therefore mostly healthier patients and better biological profile would remain in the cohort with extended follow-up.

### Strengths and limitations

Our study including a representative sample size (*n* = 420) of patients with CKD, has provided for the first time, evidence on the trajectory of kidney function and some major risk factor over time in newly referred patients in SSA using linear regression model. An assessment of the rate of decline of renal function is important but complex because renal decline is rarely a linear phenomenon and there is no gold standard methodology. A major limitation to this study is the retrospective nature of data collection with missing follow up data for some risk markers that could have influenced our results. Also proteinuria a major progression factor for CKD was not included as follow up data in this study. We could not evaluate the association between kidney function over time with onset of ESKD and mortality. Also patient on dialysis were excluded. We did not have data on treatments uptake and changes during follow-up, and therefore, could not fully account for their possible effect on the trajectories of investigated markers. Lastly, the current study was not based on power calculation. However, we used the totality of eligible observations at our center during the study period.

## Conclusion

In conclusion, our study provides additional evidence that patients with CKD in African settings are referred late to nephrologists, with an advanced stage of CKD. This likely translates into a less beneficial effect of specialised care on the course of the disease, with kidney function inexorably declining further within the first year of follow-up, regardless of underlying co-morbidities and risk factors. To what extent this progression reflects non-optimal uptake of CKD modifying therapies at baseline, and their intensification during follow-up, need to be investigated in future studies. Such information is needed to optimise the prevention of CKD progression in this setting.

## Data Availability

Data are available from the corresponding Author.

## Abbreviations

CKD: Chronic Kidney Disease
eGFR: Estimated Glomerular Filtration Rate
ESKD: End Stage Kidney Disease
RRT: Renal Replacement Therapy
CVD: Cardiovascular Disease
SSA: Sub-Saharan Africa
DGH: Douala General Hospital
MDRD: Modification of Diet in Renal Disease
KDIGO: Kidney Disease Improving Global Outcome
SBP: Systolic Blood Pressure
DBP: Diastolic Blood Pressure
